# SERPINB3 Delays Glomerulonephritis and Attenuates the Lupus-Like Disease in Lupus Murine Models by Inducing a More Tolerogenic Immune Phenotype

**DOI:** 10.3389/fimmu.2018.02081

**Published:** 2018-09-11

**Authors:** Mariele Gatto, Roberto Luisetto, Anna Ghirardello, Laura Cavicchioli, Gaia Codolo, Alessandra Biasiolo, Giuseppe Maggioni, Francesca Saccon, Marianna Beggio, Andrea Cappon, Roberta Venturini, Patrizia Pontisso, Andrea Doria

**Affiliations:** ^1^Rheumatology Unit, Department of Medicine (DIMED), University of Padova, Padova, Italy; ^2^Department of Surgery, Oncology and Gastroenterology, University of Padova, Padova, Italy; ^3^Department of Comparative Biomedicine and Food Science, University of Padova, Padova, Italy; ^4^Department of Biology, University of Padova, Padova, Italy; ^5^Internal Medicine and Hepatology Unit, Department of Medicine (DIMED), University of Padova, Padova, Italy; ^6^Laboratory Medicine Unit, University-Hospital of Padova, Padova, Italy

**Keywords:** systemic lupus erythematosus, SERPINB3, survival, lupus nephritis, apoptosis, Treg

## Abstract

**Objective:** To explore the effects of SERPINB3 administration in murine lupus models with a focus on lupus-like nephritis.

**Methods:** 40 NZB/W F1 mice were subdivided into 4 groups and intraperitoneally injected with recombinant SERPINB3 (7.5 μg/0.1 mL or 15 μg/0.1 mL) or PBS (0.1 mL) before (group 1 and 2) or after (group 3 and 4) the development of proteinuria (≥100 mg/dl). Two additional mice groups were provided by including 20 MRL/*lpr* mice which were prophylactically injected with SERPINB3 (10 mice, group 5) or PBS (10 mice, group 6). Time of occurrence and levels of anti-dsDNA and anti-C1q antibodies, proteinuria and serum creatinine, overall- and proteinuria-free survival were assessed in mice followed up to natural death. Histological analysis was performed in kidneys of both lupus models. The Th17:Treg cell ratio was assessed by flow-cytometry in splenocytes of treated and untreated MRL/*lpr* mice. Statistical analysis was performed using non parametric tests and Kaplan-Meier curves, when indicated.

**Results:** Autoantibody levels and proteinuria were significantly decreased and time of occurrence significantly delayed in SERPINB3-treated mice vs. controls. In agreement with these findings, proteinuria-free and overall survival were significantly improved in SERPINB3-treated groups vs. controls. Histological analysis demonstrated a lower prevalence of severe tubular lesions in kidneys of group 5 vs. group 6. SERPINB3-treated mice showed an overall trend toward a reduced prevalence of severe lesions in both strains. Th17:Treg ratio was significantly decreased in splenocytes of MRL/*lpr* mice treated with SERPINB3, compared to untreated control mice.

**Conclusions:** SERPINB3 significantly improves disease course and delays the onset of severe glomerulonephritis in lupus-prone mice, possibly inducing a more tolerogenic immune phenotype.

## Introduction

Development of systemic lupus erythematosus (SLE) is driven by several immunological abnormalities, including altered function of normally protective molecules and deregulated apoptosis (1, 2).

SERPINB3 belongs to SERPINs (SERin Protease INhibitors) superfamily, a group of highly conserved proteins with enzymatic anti-protease activity orchestrating organ development, cell survival and broad tissue homeostasis (3, 4). Despite its acronym, SERPINB3 is a cystein protease inhibitor whose expression is physiologically broad among epithelial tissues in the body and is deregulated in a variety of epithelial squamous and nonsquamous carcinomas, where it is involved in enhanced cell survival (5). Indeed, SERPINB3 confers resistance to apoptosis induced by different stimuli, including ultraviolet radiation (6), chemotherapeutic drugs (7) and tumor necrosis factor (TNF)-alpha (8). The prominent mechanism of inhibition is thought to be upstream caspase 3 activation, preventing cytochrome c release from mitochondria in response to oxidative stress conditions (9). Recent findings indicate that a fraction of SERPINB3 binds respiratory Complex I in the inner mitochondrial compartments, inhibiting reactive oxygen species (ROS) generation and hindering the opening of the permeability transition pore, a point of no return in cell commitment to death (7).

In addition, decreased phosphorylation of the proapoptotic molecule p38 MAPK (mitogen-activated protein kinase) or of the active forms MKK3/6 was shown in engineered 293T cells overexpressing SERPINB3, thus suggesting an additional mechanism of apoptosis inhibition (6). Importantly, the antiapoptotic potential of SERPINB3 is at least in part independent on its anti-cystein protease activity, as other kinds of kinases are still inhibited by SERPINB3 binding (10). On the other hand, SERPINB3 was found to promote endoplasmic reticulum-stress induced apoptosis, thus exerting a double-faced effect on cell viability (11).

SERPINB3 was shown to be expressed on CD27+ B lymphocyte surface of healthy donors, while it was absent on CD27+ cells of lupus patients (12). Interestingly, its expression can be down-regulated by interferon alpha (IFNα) which is up-regulated in SLE (12). Moreover, SERPINB3 determines increased production of transforming growth factor (TGF)-β and down-regulation of miR-146b-5p miRNA, implicated in TGF-β pathway inhibition (13, 14), thus suggesting a globally anti-inflammatory activity.

Deregulated apoptosis and impaired removal of cellular debris are known to play a role in SLE and lupus nephritis. Therefore, decreased or absent expression of a molecule deeply involved in the modulation of cell viability and in immune modulation represented the rationale to explore the effects of SERPINB3 restoration in murine models of SLE.

## Materials and methods

### Animal models and study design

#### New zealand black/white (NZB/NZW) F1 mice

New Zealand Black/White (NZB/NZW) F1 mice, a well characterized model of lupus-like glomerulonephritis, were treated with recombinant SERPINB3, produced as previously described (15), either before (preventive approach) or after (therapeutic approach) the development of proteinuria.

##### Preventive approach

Twenty 12-week-old NZB/NZW F1 female mice (Harlan Laboratories, Envigo RMS, UD, Italy) were subdivided into 2 groups of 10 mice each and intraperitoneally injected with a total volume of 100 μl consisting of 7.5 μg of SERPINB3 in 100 μl of phosphate buffered saline (PBS, vehicle) (group 1) or 100 μl of vehicle (group 2). Mice were injected twice a week, starting from the 17th week of age, and bred until natural death, except three mice from both groups which were sacrificed at week 27. Kidneys of mice sacrificed at week 27 (3 belonging to group 1 and 3 to group 2) were harvested for histological comparison.

##### Therapeutic approach

Twenty 12-week-old NZB/NZW F1 female mice were subdivided into 2 groups of 10 mice each and were intraperitoneally injected with a total volume of 100 μl consisting of 15 μg of SERPINB3 in 100 μl of vehicle (group 3) or 100 μl of vehicle alone (group 4). Mice were injected twice a week, starting after development of proteinuria ≥100 mg/dl, and bred until natural death.

Urine samples were collected from all mice and proteinuria was evaluated weekly.

Blood samples were collected from the caudal vein at the 17th, 21st, 25th, 28th, 32rd week of age, and at death.

Kidneys from 6 mice were harvested at death for histological examination, but were not included in the comparative analysis.

#### MRL/*lpr* mice

MRL/*lpr* mice display an impaired Fas function due to a recessive autosomal mutation named *lpr* (standing for lymphoproliferation). Descending abnormalities in the apoptotic process lead to diverse clinical features, mostly depending on dysregulated CD4+ T cell and B cell function including widespread lymphadenopathy with double negative T cell infiltrates increasing with disease severity, early severe proliferative nephritis leading to death between 3 and 7 months of age, severe necrotizing arteritis, neuropsychiatric symptoms and erosive polyarthritis (16).

MRL/*lpr* mice were treated with recombinant SERPINB3 before the development of proteinuria in order to explore the preventive approach in a multiorgan system.

Twenty 8-week-old MRL/*lpr* female mice (Harlan Laboratories) were subdivided into 2 groups of 10 mice each and were intraperitoneally injected with a total volume of 100 μl consisting of 7.5 μg of SERPINB3 in 100 μl of vehicle (group 5) or 100 μl of vehicle (group 6), as controls. Mice were injected twice a week, starting from the 9th to the 18th week of age.

Urine samples were collected and proteinuria was evaluated weekly.

Blood samples were collected from the caudal vein 3 weeks apart, starting from the 9th week of age, until mice sacrifice at week 13 (6 mice) and 16–18 (6 mice). Time for sacrifice was chosen *a priori*, taking into account the age at which the disease is known to be full-blown (around 4 months).

Kidneys were harvested from 12 mice at sacrifice for histological analyses.

This study was approved by the National Institutional Animal Care and Use Committee.

### Recombinant SERPINB3 synthesis

SERPINB3 cDNA was obtained as previously described (15). Briefly, first strand cDNA was synthesized by nested polymerase chain reaction (PCR), using 2 pairs of specific oligonucleotide primers (Invitrogen - Primer out Forward 5′-CACAGGAGTTCCAGATCACATCGAG-3′ Primer out Reverse 5′-CTGGAAGAAAAAGTACATTTATATGTGGGC-3′ Primer out Forward 5′-CACCATGAATTCACTCAGTGAAGCCA-3′ Primer out Reverse 5′-ATTGCATCTACGGGGATGAG-3′). The obtained PCR fragment was inserted into mammalian expression vectors and propagated into E. Coli competent cells. Recombinant SERPINB3 protein was characterized on retained dialyzed and cation-exchange chromatography purified soluble fraction from crude bacterial extract by SDS-PAGE with Coomassie blue staining (15). To remove endotoxins, the recombinant SERPINB3 protein was loaded into a 1 mL high-capacity endotoxin removal spin column (Pierce Biotechnology, Rockford IL, USA) following the manufacturer's protocol. The processed protein was collected by centrifugation at 500 × g for 5 min in the endotoxin-free tube. Endotoxin levels were assessed using Endosafe portable test system (Charles River Laboratories) based on the Limulous amoebocyte lysate (LAL) method. The sensitivity of the assay was 0.05 EU/mL and SERPINB3 preparation used in the study had undetectable endotoxin levels.

### Evaluation of clinical variables: proteinuria, serum creatinine, and survival

Disease progression was monitored by weekly urine sample collection, in order to evaluate proteinuria levels, using multi-reactive strips (Siemens) and expressed as mg/dl. Proteinuria was evaluated according to the manufacturer: negative, slight positive, positive: + = 30 mg/dl, ++ = 100 mg/dl, +++ = 300 mg/dl, and ++++ ≥2,000 mg/dl albumin. Traces of proteinuria were defined as 15 mg/dl.

Renal function was monitored through serum creatinine levels (μmol/L) that were evaluated on blood samples withdrawn every 3 weeks starting from week 9 in MRL/*lpr* mice and from week 17 in NZB/W F1 mice. Creatinine assessment was carried out on Cobas 8000 (Roche Diagnostics) using an enzymatic method, traceable to Isotope Dilution Mass Spectrometry (IDMS) reference procedure.

Disease-free and overall survival were evaluated in all mice. Proteinuria-free survival was defined as <300 mg/dl, according to manufacturer's instruction, as the threshold of 300 mg/dl designates a frank positivity.

### Measurement of serum autoantibodies

Serum levels of mouse IgG anti-C1q and anti-dsDNA antibodies were evaluated by standardized home-made ELISA tests as previously described (17).

Briefly, for anti-C1q antibodies, plates were coated with C1q at a concentration of 5 μg/ml. Sera were added in duplicate diluted 1:4 in 1% BSA/PBS with 1 M NaCl, to prevent immunocomplexes (ICs) formation. Alkaline phosphatase-conjugated goat anti-mouse IgG was added at the dilution of 1:10,000 in 1% BSA/PBS with 1 M NaCl. Finally, *p*-nitrophenyl phosphate was added. The plates were read at 405 nm and optical density (OD) 0.287 was chosen as cut-off value. For anti-dsDNA antibodies, plates were coated following 3 steps: addition of poly-L-lysin at a concentration of 10 μg/ml, in order to catch the DNA; then addition of calf fetal dsDNA at a concentration of 25 μg/ml; finally, addition of poly-L-glutamate at a concentration of 5 μg/ml in order to neutralize the free negative charges of DNA. Sera were added in duplicates diluted 1:100 in 1% BSA/TBS. Alkaline phosphatase-conjugated goat anti-mouse IgG was added at the dilution of 1:10,000 in 1% BSA/TBS. Finally, *p*-nitrophenyl phosphate was added. The plates were read at 405 nm and OD 0.233 was chosen as cut-off value. Since a calibrator for murine anti-C1q and anti-dsDNA autoantibody quantification is not commercially available nor feasibly realizable, autoantibody levels were expressed as OD units.

ELISA reagents were purchased from Sigma, St Louis, USA; ELISA plates were purchased by Nalgene Nunc, New York, USA.

### Histopathological analysis

Six NZB/WF1 mice sacrificed at week 27 underwent histological comparison (three from group 1 and three from group 2). Kidneys of 12 out of 20 Mrl/lpr mice were harvested for histological analysis and comparison; six out of 12 mice analyzed had been sacrificed at week 13 and 6 at week 16–18.

Renal sections as thin as 3 μm (microtome Leica RM 2145) underwent histopathological evaluation by optical microscopy, focusing on renal functional compartments (glomeruli, tubuli, interstitium, vessels). A minimum of 8 glomeruli per sample was required.

In order to properly evaluate different renal compartments, specific histological stainings were used. Samples were fixed in formalin 10% and embedded in paraffine and then stained by Hematoxyline-Eosin (HE), Periodic Acid Schiff (PAS), Acid Fuchsin Orange G (AFOG), Masson Trichrome staining (TRIC) and Periodic Acid Schiff Methanamine (PASM).

Renal sections were then screened by experienced pathologists in a blinded fashion for presence of glomerular lesions (glomerulosclerosis and mesangial hyperplasia), perivascular inflammation and tubular lesions (tubular dilation and presence of casts), according to a semiquantitative score from 0−1 (no/mild involvement) to 4–5 (very high involvement), as detailed in Supplementary Table 1 [courtesy of Prof. Susan Westmoreland, DVM-*ABBVIE Pharmaceuticals*; IL, USA and (18)].

### Isolation of T lymphocytes from MRL/*lpr* mice

SERPINB3-treated and vehicle-treated MRL/*lpr* mice were sacrificed at 13 and 16–18 weeks of age. The spleens were removed and dissociated in RPMI medium supplemented with 50 mM HEPES and 10% fetal bovine serum. The cell suspension was passed through a 70-μm strainer, and cells were collected by centrifugation at 300 *g* for 5 min. Erythrocytes were lysed by incubating the cells in red blood cell lysis buffer (eBiosciences) at room temperature for 5 min. T lymphocytes were then isolated using the EasySep™ mouse T cells isolation kit (StemCell™ technologies) following the manufacturer's instructions.

### Flow cytometry analysis

CD4 + CD25 + Foxp3 + regulatory T (Treg) cells are important regulators of immune response and the imbalance between Treg cells and T helper (Th)17 cells has been already described in a number of different inflammatory and autoimmune diseases (19).

The Th17:Treg ratio in T cells isolated from spleen of SERPINB3-treated and vehicle-treated MRL/*lpr* mice was determined by flow cytometry analysis. Single-cell suspensions were prepared from the collected splenocytes and incubated with APC-conjugated anti-mouse CD3, peridinin-chlorophyll proteins (PerCP Cy5.5)-conjugated anti-mouse CD4, phycoerythrin (PE)–conjugated anti-mouse CD25, FITC–conjugated anti-mouse FoxP3, PE-conjugated anti-mouse interleukin (IL)-17, or their respective isotype controls, for evaluation of T cell subsets. Staining for FoxP3 was conducted using an eBioscience FoxP3 staining kit. Cells were washed and analyzed using FACSCanto system (Becton Dickinson Immunocytometry Systems).

For detection of Th17 cells, T cells (1 × 10^6^ cells/well) were incubated with 50 ng/ml phorbol myristate acetate and 1 μg/ml ionomycin (Sigma) in the presence of brefeldinA 5 μg/ml (Sigma) for 5 h, before intracellular staining. Analyses were performed using FlowJo software (Tree Star).

Data were expressed as the % mean of positive cells ± SD (standard deviation).

### Statistical analyses

The ELISA results for all autoantibodies were expressed as mean OD of the duplicates of each serum. The optimal antibody cut-off values for anti-C1q and anti-dsDNA were calculated as mean OD plus 1 SD of the samples of mice before the development of traces of proteinuria (15 mg/dl). Autoantibody levels were expressed as median (min-max) of the mean OD of the double of every serum.

The differences between groups were analyzed using Mann-Whitney U test for nonparametric continuous variables; proteinuria-free survival rate (proteinuria <300 mg/dl) and survival rates were evaluated by Kaplan-Meyer method using Mantel-Cox test for comparison. Analysis of covariance during time was calculated by ANOVA for repeated measures in MRL/*lpr* mice sacrificed at discrete time points. Within-group contrasts were performed according to Bonferroni's method. Results of ANOVA are presented reporting 3 F test values, namely F_G_ for grouping factor, F_T_ for time, and F_GxT_ for the interaction between time and grouping factors. Chi-squared test was used for histological comparison. Flow cytometry data were analyzed by Student's *t*-test for independent samples. IBM SPSS Statistics 22 for Windows software (IBM SPSS Inc., USA) was used for calculations. A *p* value ≤ 0.05 was considered statistically significant.

## Results

### Autoantibody serum levels

#### Preventive SERPINB3 treatment in NZB/NZW F1 mice

**Figure 1:**
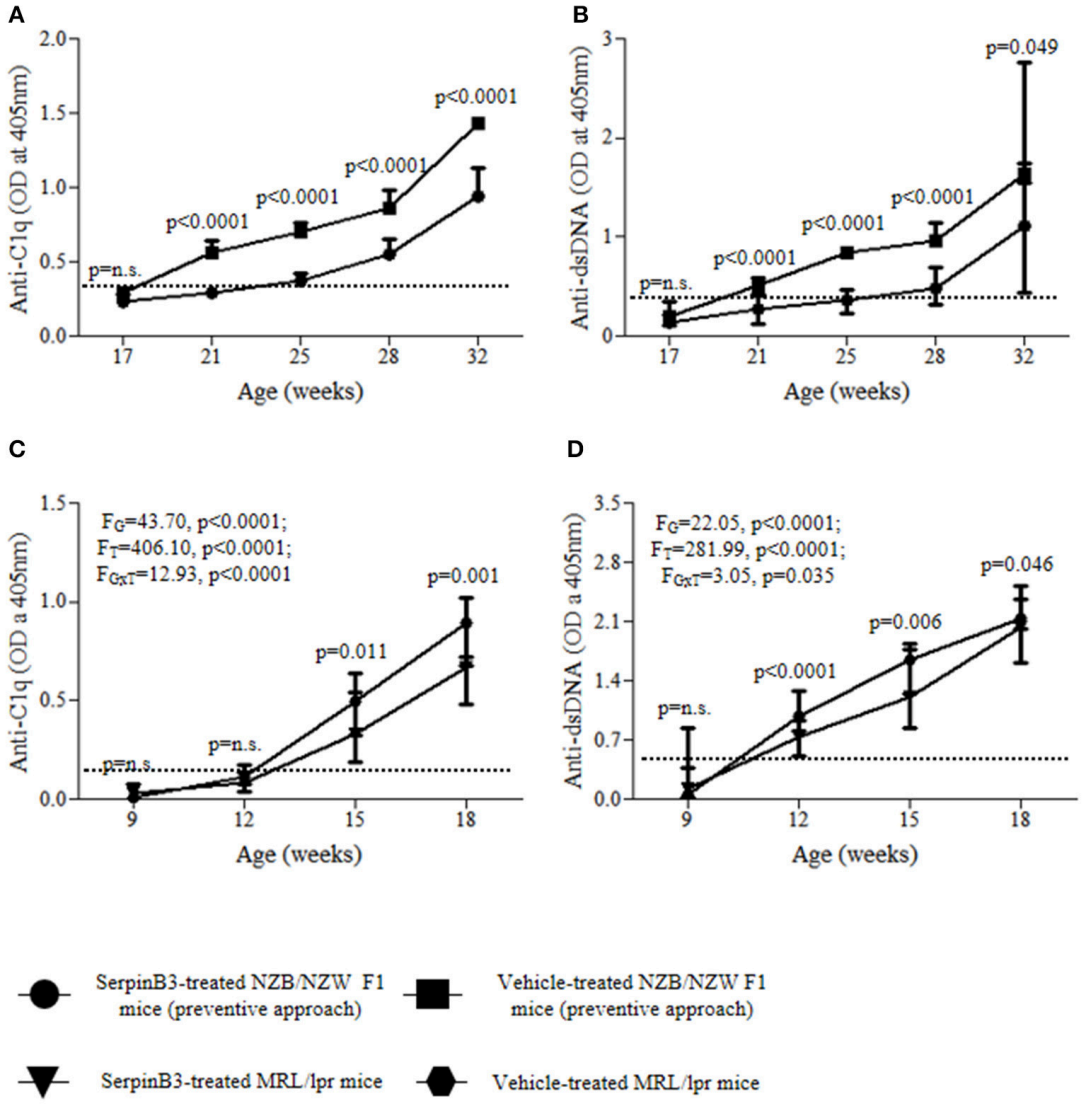
Serum autoantibody levels in SERPINB3-treated and vehicle-treated mice: anti-C1q **(A)** and anti-dsDNA **(B)** in NZB/NZW F1 mice (preventive approach); anti-C1q **(C)** and anti-dsDNA **(D)** in MRL/*lpr* mice. Serum dilution for anti-C1q antibody determinazion: 1:4; serum dilution for anti-dsDNA antibody determination: 1:100. The dotted-lines represent the respective antibody cut-off levels. The values are expressed as median (min-max) OD for autoantibodies. Anti-C1q, antibodies directed to the complement C1q component; Anti-dsDNA, antibodies directed to the double stranded DNA; OD, optical density.

SERPINB3-treated mice (group 1) developed anti-C1q antibodies significantly later and at lower levels than control mice (group 2). Anti-C1q antibodies were detected in mice of group 1 starting from the 25th week of age, while in group 2 from the 21st week of age (Figure 1A). The difference between group 1 and group 2 was statistically significant at weeks 21, 25, 28, and 32 (*p* < 0.0001 for all time points).

A similar course was observed for anti-dsDNA antibodies, as mice of group 1 developed anti-dsDNA later and at significantly lower levels than group 2. Anti-dsDNA antibodies were detected in group 1 starting from the 28th week of age, while in group 2 they became detectable from the 21st week of age (Figure 1B). The difference between group 1 and group 2 was statistically significant at weeks 21, 25, and 28 (*p* < 0.0001 for all time points).

#### Preventive SERPINB3 treatment in MRL/*lpr* mice

SERPINB3-treated MRL/*lpr* mice (group 5) developed anti-C1q antibodies significantly later and at lower levels than control mice (group 6) (*F*_G_ = 43.70, *p* < 0.0001; *F*_T_ = 406.10, *p* < 0.0001; F_GxT_ = 12.93, *p* < 0.0001). Anti-C1q antibodies were detected in mice of group 5 starting from the 15th week of age, while in group 6 from the 12th week of age (Figure 1C). The difference between group 5 and group 6 was statistically significant at weeks 15 (*p* = 0.011) and 18 (*p* = 0.001).

Mice of group 5 developed anti-dsDNA antibodies later and at lower levels than mice of group 6 (*F*_G_ = 22.05, *p* < 0.0001; *F*_T_ = 281.99, *p* < 0.0001; F_GxT_ = 3.05, *p* = 0.035; Figure 1D). Indeed, while at week 12 anti-dsDNA antibodies were detectable in all mice from group 6, they occurred only in one mouse from group 5. The difference between group 5 and group 6 was statistically significant at weeks 12 (*p* < 0.0001), 15 (*p* = 0.006), and 18 (*p* = 0.046).

#### Therapeutic SERPINB3 treatment in NZB/NZW F1 mice

**Figure 2:**
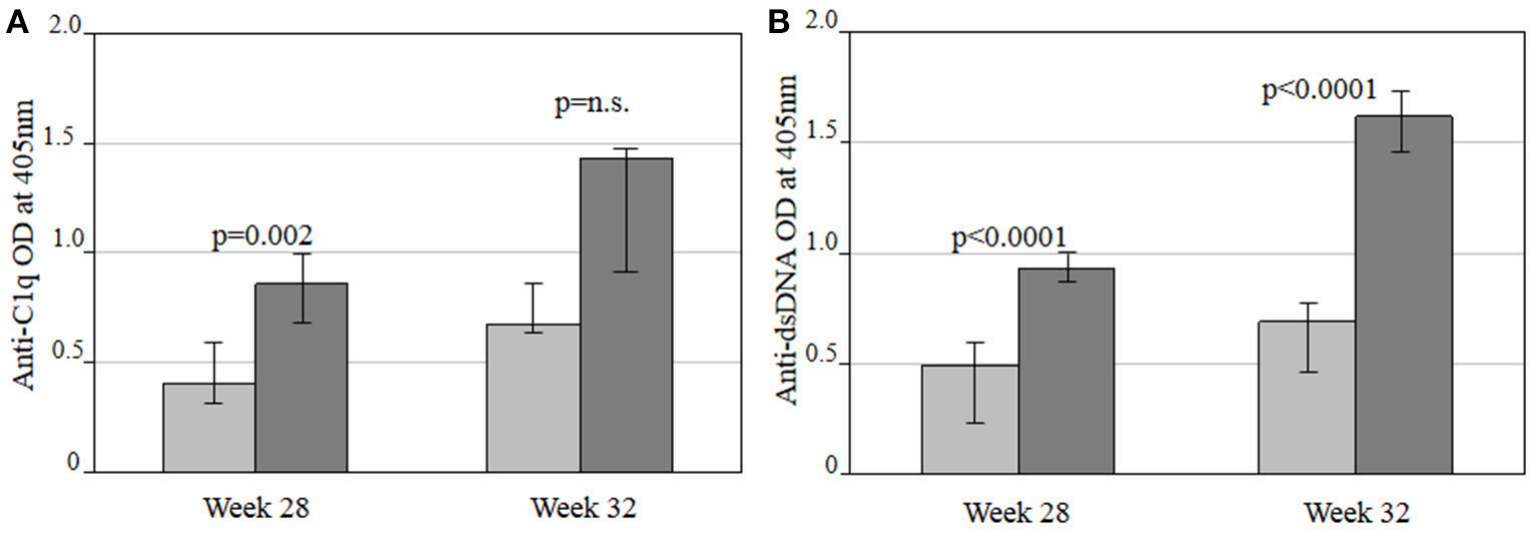
Comparison of anti-C1q **(A)** and anti-dsDNA **(B)** autoantibody levels between SERPINB3-treated (light gray bars) and vehicle-treated NZB/NZW F1 (dark gray bars) mice in the therapeutic approach at 28 and 32 weeks of treatment, respectively. Antibody levels are expressed as the median (min-max) of the mean OD of the double of every serum. OD, optical density; Anti-C1q, antibodies against complement fragment 1; Anti-dsDNA, antibodies against double stranded DNA.

Anti-C1q and anti-dsDNA antibody levels were lower in SERPINB3-treated NZB/NZW F1 mice (group 3) vs. control mice (group 4), with significant differences in median OD at week 28 for anti-C1q (*p* = 0.002) and at weeks 28 and 32 for anti-dsDNA antibodies (*p* < 0.0001 for both) as shown in Figures 2A,B.

### Renal variables, proteinuria-free, and global survival rates in mice

Proteinuria levels were expressed in mg/dl as median (min-max) and overall results are reported in Table 1.

**Table 1:** Comparison of proteinuria between SERPINB3-treated and vehicle-treated NZB/NZW F1 mice (preventive and therapeutic approaches) and MRL/*lpr* mice.

**NZB/W F1 mice**	**SERPINB3-treated**	**Vehicle-treated**	***p***
**PREVENTIVE APPROACH**			
Week 17	0 (0–0)	0 (0–0)	n.s.
Week 21	0 (0–0)	15 (0–15)	0.023
Week 23	0 (0–0)	15 (0–2000)	0.001
Week 25	15 (0–100)	23 (15–2000)	n.s.
Week 27	30 (15–300)	2000 (15–2000)	n.s.
Week 29	65 (15–300)	2000 (100–2000)	0.036
Week 33	300 (30–2000)	2000 (300–2000)	n.s.
**THERAPEUTIC APPROACH**			
Week 17	0 (0–0)	0 (0–0)	n.s.
Week 21	0 (0–15)	0 (0–0)	n.s.
Week 23	30 (15–30)	15 (0–15)	0.010
Week 25	30 (30–100)	15 (0–300)	0.045
Week 27	100 (100–100)	15 (0–300)	n.s.
Week 29	100 (15–2000)	165 (15–2000)	n.s.
Week 33	30 (15–2000)	300 (100–2000)	n.s.
**MRL/*****lpr*** **MICE (PROPHYLACTIC APPROACH)**			
w9	0 (0–0)	0 (0–0)	n.s.
w12	15 (0–30)	100 (15–100)	0.001
w15	100 (30–300)	100 (100–300)	n.s.
w18	100 (100–2000)	300 (300–2000)	0.008

*Proteinuria is expressed in mg/dl as median (min-max). NZB/WF1, New Zealand Black/White first generation; n.s., not significant*.

#### Preventive SERPINB3 treatment in NZB/NZW F1 mice

SERPINB3-treated mice (group 1) developed proteinuria later and at lower levels than control mice (group 2). Indeed, 6 (60%) mice in group 1 developed traces of proteinuria at the 25th week of age and the remaining 4 at the 27th week of age, whereas all mice from group 2 developed proteinuria within week 25. At the 27th week of age, 10% of group 1 mice showed proteinuria levels ≥300 mg/dl vs. 90% of group 2 mice. Difference of proteinuria between group 1 and group 2 was significant at weeks 21 (*p* = 0.023), 23 (*p* = 0.001), and 29 (*p* = 0.036), as shown in Table 1.

**Figure 3:**
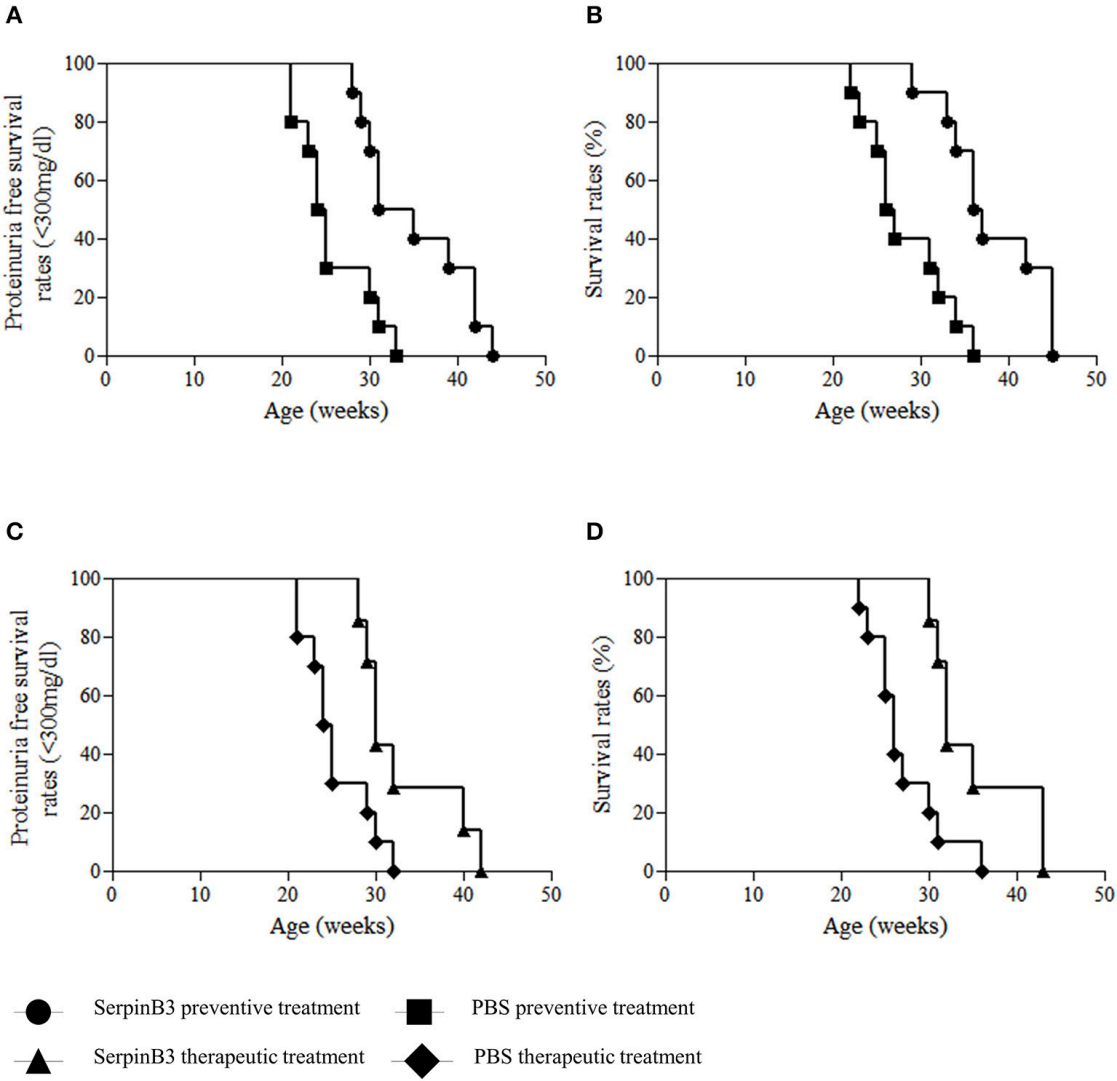
Proteinuria free survival rates (<300 mg/dl) and overall survival rates in SERPINB3-treated and vehicle-treated NZB/NZW F1 mice in the preventive approach **(A,B)** and in the therapeutic approach **(C,D)**.

Proteinuria-free survival rate (proteinuria <300 mg/dl) was significantly higher in group 1 than in group 2 mice (*p* = 0.002) (Figure 3A). At the 31st week of age, 50% of mice in group 1 had proteinuria levels ≥300 mg/dl, compared with 90% in group 2; the last mouse of group 1 developed proteinuria ≥300 mg/dl at the 44th week of age and the last of group 2 at the 33rd week of age (Figure 3A).

Accordingly, global survival rate was significantly higher in group 1 than in group 2 mice (*p* = 0.001) (Figure 3B). In group 2, 50% of mice died within the 26th week of age while none of SERPINB3-treated mice died within this time frame. At the 36th week of age, 50% of group 1, but none of the mice of group 2, were still alive. The last deaths in the SERPINB3-treated group occurred in three 45-week-old mice (Figure 3B).

Notably, all mice brought to natural death died after developing the maximum detectable levels of proteinuria (≥300 mg/dl).

No significant difference was found at any time point in creatinine serum levels between groups (data not shown).

#### Therapeutic SERPINB3 treatment in NZB/NZW F1 mice

Mice were treated between week 24 and 27, i.e., after occurrence of proteinuria≥100 mg/dl. At the 25th week of age, 40% of mice of group 3 (SERPINB3 treated) showed proteinuria levels ≥300 mg/dl vs. 100% of mice of group 4 (controls). Proteinuria median levels were lower in group 3 than in group 4 mice starting from week 29, despite not reaching statistical significance.

Proteinuria-free survival rate (proteinuria <300 mg/dl) was significantly higher in group 3 than in group 4 (*p* = 0.014) (Figure 3C). At the 25th week of age, none of the mice in group 3 had proteinuria levels ≥300 mg/dl, compared with 50% of mice in group 4. The last mouse from group 3 developed proteinuria ≥300 mg/dl at the 42nd week of age and the last of group 4 at the 32nd week of age (Figure 3C).

Global survival rate was significantly higher in group 3 than in group 4 mice (*p* = 0.009) (Figure 3D). In group 4, 60% of mice died within the 26th week of age vs. 0 of SerpinB3-treated mice. At the 36th week of age, 30% of group 3 mice, but none of the mice of group 4, were still alive. The last 2 mice in the SERPINB3-treated mice died at the 45th week of age (Figure 3D).

Also in this case, all mice died after developing the maximum detectable levels of proteinuria (≥300 mg/dl) and no significant difference between groups was found in creatinine serum levels at any time point (data not shown).

#### Preventive SERPINB3 treatment in MRL/*lpr* mice

SERPINB3-treated mice (group 5) developed proteinuria later and at lower levels than control mice (group 6) (*F*_G_ = 3.93, *p* < 0.0001; *F*_T_ = 10.04, *p* < 0.0001; F_GxT_ = 2.32, *p* = 0.084). Difference between group 5 and group 6 was significant at weeks 12 (*p* = 0.001) and 18 (*p* = 0.008) (Table 1). Indeed, 3 (30%) mice from group 5 developed traces of proteinuria starting from the 12th week of age, whereas all mice of group 6 developed traces of proteinuria within this week. At the 15th week of age, only 1 (10%) mouse of group 5 showed proteinuria levels ≥300 mg/dl vs. 3 (30%) of group 6. At the time of programmed sacrifice at week 16–18 only 2 (20%) mice of group 5 developed proteinuria ≥300 mg/dl vs. 100% of control mice.

No significant difference was found at any time point in creatinine serum levels between groups (data not shown).

### Histological analysis

Both mice strains develop a lupus-like nephritis.

Only organs harvested from mice sacrificed at given timepoints were included in the analysis.

Actual scores of histological lesions among sacrificed littermates are reported in Supplementary Table 2.

Among MRL/*lpr* mice sacrificed at week 13, no significant difference was found in glomerular, tubular or interstitial lesions between treated and control mice, though the percentage of mice showing severe glomerular lesions (score 5) was higher in group 6 vs. group 5 (33% vs. 0).

Among MRL/*lpr* mice sacrificed at week 16–18, severe tubular lesions (score 4) were found to be significantly more prevalent in group 6 vs. group 5 (*p* = 0.014). Severe glomerular lesions were found to be comparable in group 5 vs. group 6. No difference was found in severity of perivascular inflammation.

Concerning NZB/W F1 mice sacrificed at week 27, prevalence of severe glomerular and tubular lesions were comparable among group 1 and group 2.

**Figure 4:**
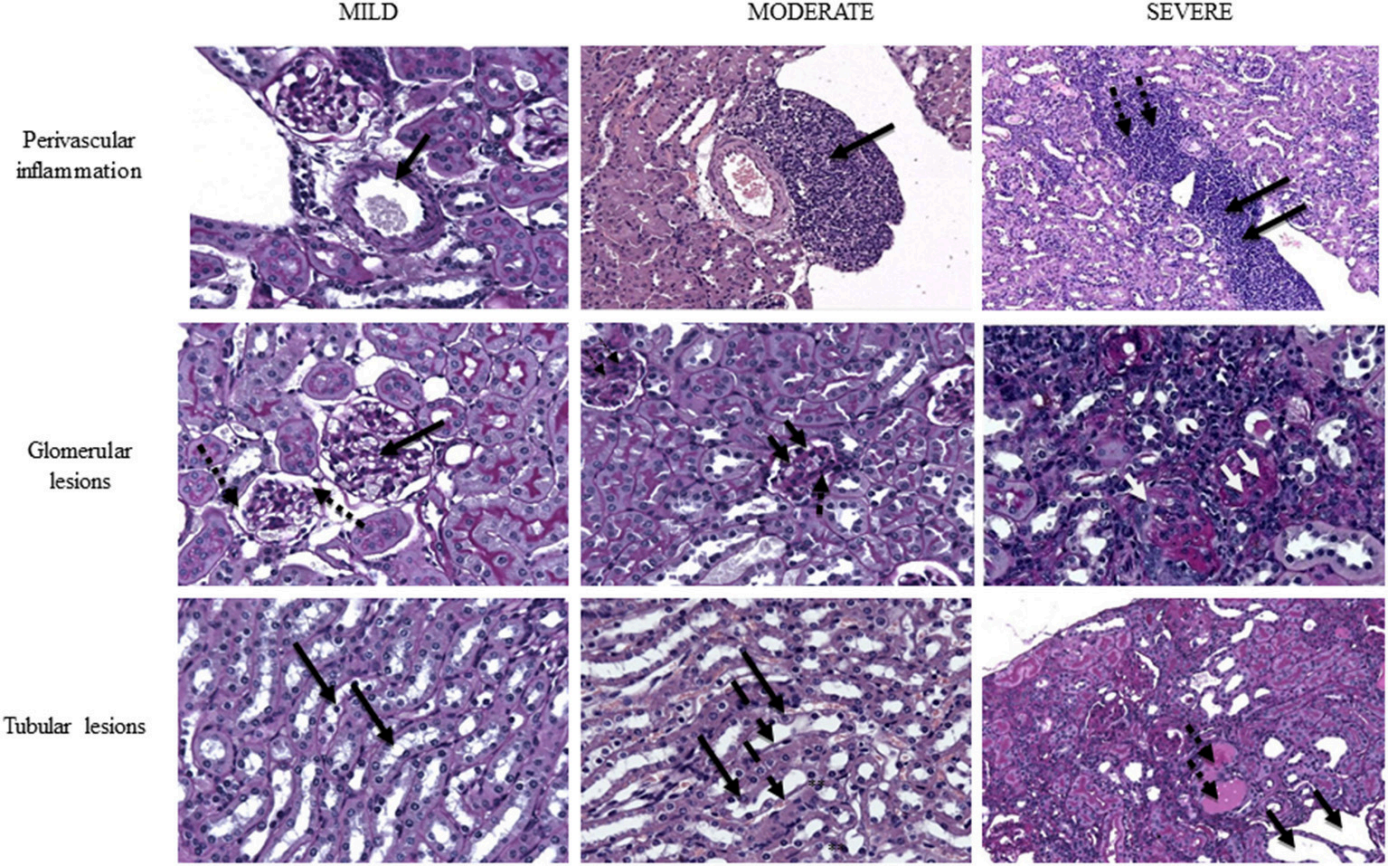
Representative images of mouse kidney with different extent of perivascular lesions (upper row), glomerular lesions (middle row) and tubular lesions (lower row). **Upper** row, left to right: normal vasculature with patent artery and clear renal vein (magnification PAS40x), indicated by thick arrow; grade 3 infiltrate partially surrounding the renal artery and bulging into the adjacent vein with sparing of connective tissue (HE20x), indicated by thick arrow; grade 5 perivascular infiltrate fully surrounding the arcuate artery and invading both the vein (thick black arrows) and the adjacent connective tissue (thick dotted black arrows) (HE10x). **Middle** row, left to right: near normal glomeruli (thick black arrows) with preserved basement membrane and urinary space (thick dotted black arrows) (PAS40x); intermediate damaged glomeruli with reduced urinary space, thickened basement membrane (thick black arrows), cellular hyperplasia (thick dotted black arrows) and initial septal fibrosis (thin dotted black arrows) (PAS40x); advanced glomerular damage with disappearance of glomerular structure, glomerular collapse and diffuse glomerulosclerosis (two white thick arrows; single arrows pinpoints absence of urinary space due to collapse). Diffuse lymphocytic infiltrate is also present (PAS40x). **Lower** row, left to right: near normal renal tubuli at the medullo-cortical interface with normal cell nuclei in a wide cytoplasm (thick black arrows) (PAS40x); initial tubular dilation with flattened epithelial cells (thick dashed black arrows) with nuclei bulging into the tubular space (thick black arrows) (magnification PAS40x); severely dilated tubuli (thick black arrows) with loss of normal architecture and widely represented protein casts (pink lakes) (thick dotted black arrows) (PAS10x). PAS, periodic acid of Schiff; HE, hematoxylin-eosin.

Figure 4 shows representative kidney images with various degrees of severity.

### SERPINB3 increases treg cells in MRL/*lpr* splenocytes

A remarkable increase in Treg cells was observed in T lymphocytes isolated from spleen of SERPINB3-treated MRL/*lpr* mice that was significantly higher than in controls at week 13 (% mean ± SD: week 13: serpin 2.1%± 0.05 vs. PBS 1.4% ± 0.01, *p* < 0.0001 Week 16: serpin 5.2 ± 0.7 vs. PBS 3.2 ± 0.8).

**Figure 5:**
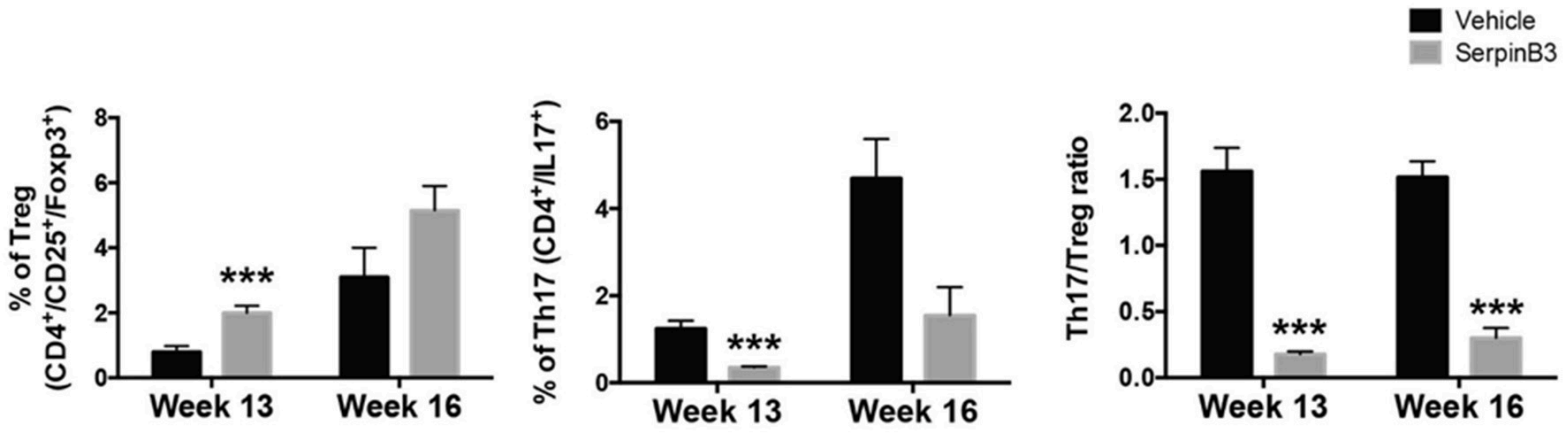
Left to right. Proportion of Treg on total pool of CD4+, week 13 to week 16. Proportion of Th17 on total pool of CD4+, week 13 to week 16. Ratio of Th17 cells to Treg cells (*n* = 3 per group). Results are the mean ± SD. ***P < 0.0001 vs. vehicle-treated controls.

These findings were associated with a parallel decrease in Th17 cell number (% mean ± SD: week 13: serpin 0.5 ± 0.01 vs. PBS 1.7 ± 0.05, p < 0.0001; week 16 serpin 1.8% ± 0.8 vs. PBS 4.8% ± 1.2), resulting in a significant reduction in the Th17:Treg cell ratio in SERPINB3-treated mice compared with PBS-treated mice (Figure 5).

## Discussion

SERPINB3 is a pleiotropic molecule found to be decreased on the surface of B lymphocytes in SLE patients, being likely down-regulated by IFN-α (12). SERPINB3 is deeply involved in the control of apoptotic cell death (11, 20), which is markedly impaired in patients with SLE (12), and was shown to up-regulate TGF-β by autocrine and paracrine pathways (13, 14, 21), hinting at a global pro-fibrogenic and anti-inflammatory effect. On the basis of these findings, the present study has been addressed to analyze the effects of restoration of SERPINB3 in murine models of SLE.

NZB/W F1 mice were chosen as a well-known model of lupus-like nephritis which allowed our experiments to focus on the kidney, as the major target organ in SLE, while MRL/*lpr* mice were chosen as additional model of systemic lupus-like disease, resembling the complexity of human disease.

Mice injected with SERPINB3 showed a remarkable clinical improvement in comparison with their control littermates, with statistically significant delay in autoantibody appearance, decrease in circulating autoantibody levels and increased proteinuria-free and overall survival, suggesting that SERPINB3 can actually improve the course of the disease.

Interestingly, improvement in clinical and serological parameters occurred in NZB/W F1 mice both with the preventive and the therapeutic approach, suggesting a protective potential of SERPINB3 even when renal disease is already established. Despite statistical significance was not reached for proteinuria difference following the therapeutic approach, which may be due to inadequate mice number, the decrease is apparent and likely clinically relevant, as suggested by the improved survival after therapeutic SERPINB3 administration.

Notably, decreased autoantibody and proteinuria levels, associated with improved survival rates, were also confirmed in MRL/*lpr* mice, thus suggesting a beneficial effect of SERPINB3 even in a heterogeneous system. As MRL/*lpr* mice are characterized by a mutation in the Fas domain, any counteracting effect on uncontrolled apoptosis may have been responsible for the observed benefits.

Although our study was focused on clinical variables and outcomes and was not designed to evaluate tissue lesions, we looked for changes in renal histology by analyzing renal sections of mice sacrificed at given time points, which were chosen according to time of autoantibody and proteinuria occurrence. Interstitial perivascular lesions were quite similar among the groups, which may be due to perivascular infiltrate appearing early during nephritis course, making it unlikely to differ in the intermediate or late stages of disease. Both mice strains treated with SERPINB3 showed milder tubular and glomerular lesions, with a lower proportion of severe tubular lesions, compared to controls. These observations may have a clinical relevance, though mice number was probably too low for statistical analysis and the scoring system itself may have overlooked small differences in disease severity. Indeed, although validated, any semiquantative score for histological lesions bears the risk of low sensitivity due to a range of values that embrace even very different degrees of severity, thus sometimes flattening actual clinical discrepancies.

Our results are in agreement with previous data obtained with another member of the clade B serpins (OV-SERPINS), namely ovalbumin (OVA), where OVA-injected NZB/NZW F1 mice showed decreased anti-dsDNA antibody titers and proteinuria levels, as well as a prolonged proteinuria-free survival (Blank et al., personal communication). Since both OVA and SERPINB3 were shown to improve the disease outcome in murine lupus models, these might conceivably play a role in maintaining immune homeostasis, and this novel function is in line with the versatility of the serpin platform, recently described involved in the regulation of diverse biological pathways (22). Furthermore, other authors reported clinical improvement of lupus-like disease and arthritis following administration of a fragment of SERPINA1 and SERPINA3, respectively, which, although belonging to different serpin subgroups, are sharing similar mechanisms of action with OV-SERPINS (23, 24).

The mechanistic link between the known functions of SERPINB3 and the improved clinical outcome observed in treated mice has to be unraveled. It is so far well known that the enrichment of the autoantigen pool due to deregulated apoptosis may foster autoantibody production in human SLE (1). Since SERPINB3 was shown to globally dampen the apoptotic pathways, absence of SERPINB3 may result in enhanced cellular turnover, with increased production and exposition of cellular debris, which may in turn stimulate the generation of autoantibodies toward nuclear antigens.

The increase of Treg cells, together with a parallel decrease of Th17 cells, observed within the T cell pool from spleens of MRL/*lpr* mice treated with SERPINB3 is a novel finding. These regulatory cells are likely peripheral Treg dwelling in tissues, which are known to have their FoxP3 expression induced by changes in the surrounding microenvironment (25). Since cytokines including TGFβ have some effects on plasticity of T cells in the periphery (26), and given that TGFβ secretion is promoted by SERPINB3 (13, 14), it may be speculated that in lymphoid organs SERPINB3 may skew cells toward a more tolerogenic phenotype. This hypothesis is in line with the finding of decreased levels of IL-17 and IL-12 in MRL/*lpr* mice treated with a fragment of SERPINA1 and in CD4+ cells incubated with the same peptide (23). These observations suggest that SERPINS, including SERPINB3, may influence the immune homeostasis through shaping of the extracellular microenvironment.

Further studies will be required to investigate actual mechanisms of action.

It should be reminded that perturbation of homeostatic pathways is often involved in the development of autoimmunity and in SLE immunomodulatory therapies targeting self-molecules and cytokines are released or underway (27–30). Actually, most of these drugs aim at reducing the hyperactivation of physiological signals (31), while the case of SERPINB3 would require the restoration of a normally activated pathway that is instead downregulated in SLE. This is a new approach and implies safety considerations.

The present study has some limitations. The number of mice included in the histological comparison was too low to draw firm conclusion on potential effects of SERPINB3 on renal lesions, which should then be included in a future outlook.

Moreover, exogenous administration of SERPINB3 to lupus-prone mice causes increased concentrations of SERPINB3 in mice sera and tissues, therefore one may claim that it does not mirror the physiological activity of SERPINB3. However, different administration routes have been exploited so far (20, 23) which do not overshadow the evidence that SERPINS administration delay the onset of lupus-like nephritis and firmly improve overall- and disease-free survival in mice.

In summary, our data have shown that exogenous SERPINB3 induces a remarkable improvement in terms of both survival and clinical outcome in lupus-prone mice. Particularly, SERPINB3-injected mice developed a milder and markedly delayed renal disease involvement. Notably, these findings were associated with later appearance of nephritogenic antibodies in sera and their levels were significantly lower than those found in controls. Therefore, present clues suggest that this molecule may skew cells in lymphoid organs toward a more tolerogenic phenotype, counteracting the onset of severe lupus glomerulonephritis and attenuating lupus phenotype.

## Author contributions

MG and AG wrote the manuscript. RL bred and sacrificed the mice harvesting the kidneys and collected blood and urine samples. MG, LC, GM, FS, and MB reviewed histological samples at light microscopy. AG performed ELISA tests and proteinuria measurements. RV performed biochemical tests on mice urine. GC and AC retrieved the splenocytes and performed FACS analysis. AB materially produced human recombinant SERPINB3. PP provided SERPINB3 and contributed to manuscript writing. AD conceived the core of the project, coordinated overall work and revised the manuscript allowing final version.

### Conflict of interest statement

The authors declare that the research was conducted in the absence of any commercial or financial relationships that could be construed as a potential conflict of interest.
