# Supplementary material for: SERPINB3 Delays Glomerulonephritis and Attenuates the Lupus-Like Disease in Lupus Murine Models by Inducing a More Tolerogenic Immune Phenotype
Source: Front Immunol. 2018 Sep 11;9:2081. doi: 10.3389/fimmu.2018.02081 (PMC6141748; doi:10.3389/fimmu.2018.02081)
Supplement: Supplementary file 1 [file Table_1.DOC]

|  | **Score** | **Glomerular lesions** | | **Tubular lesions** | | **Interstitial lesions**  **(perivascular inflammation)** |
| --- | --- | --- | --- | --- | --- | --- |
|  |  | **% of affected glomeruli** | **Severity of lesions** | **Tubular dilation** | **Casts (expressed as the number of affected nephrons)** |  |
| Mild | 0 | <2 |  |  | 0-1 | Up to a few rare lymphocytes |
|  | 1 | 2-10 | Segmental thickening of mesangium | Minimal dilation of few tubules, either as a focus or multifocal; generally limited to inner stripes of outer medulla | 2-10 | A few lymphocytes forming loose aggregates |
|  | 2 | 10-30 | Segmental to diffuse thickening of the mesangium, most glomeruli | Minimal/mild dilation of up to 20% of the tubules either as a focus or multifocal; generally limited to inner stripes of outer medulla | 11-20 | Lymphocytes forming discrete small aggregates |
| Moderate | 3 | 30-50 | Diffuse thickening of mesangium, hypercellular glomerulus, increased size of podocytes, generally no adherences | Mild/moderate dilation of up to 50% of the tubules, expands to same areas of the cortex | 20-30 | Polarized aggregates of lymphocytes that bulge into the lumen of the adjacent vein but fail to fully surround the arcuate artery |
| Severe | 4 | 50-70 | Diffuse thickening of mesangium, my be coagulated proteins or fibrous, hypocellular glomerulus, increased size of podocytes and Bowman’s capsule epithelium, adherences, crescents | Mild/moderate dilation of > 50% of the tubules, expands througout the cortex | 30-40 | Lymphocyte aggregate fully surrounding the arcuate artery and not showing obvious polarization |
|  | 5 | >70 |  |  |  | Lymphocytic infiltrate extending from the adventitia of the arcuate artery into the adjacent connective tissue |

**Supplementary Table 1. Detailed semiquantitative scoring system to quantify renal lesions**
