# Supplementary material for: SERPINB3 Delays Glomerulonephritis and Attenuates the Lupus-Like Disease in Lupus Murine Models by Inducing a More Tolerogenic Immune Phenotype
Source: Front Immunol. 2018 Sep 11;9:2081. doi: 10.3389/fimmu.2018.02081 (PMC6141748; doi:10.3389/fimmu.2018.02081)
Supplement: Supplementary file 2 [file Table_2.DOCX]

|  | **Group** | **Glomerular lesions** | | **Total glomerular score** | **Tubular lesions** | | | **Total tubular score** | **Mouse age (weeks)** |
| --- | --- | --- | --- | --- | --- | --- | --- | --- | --- |
|  |  | **% affected glomeruli** | **Mesangial hypercellularity and sclerosis** |  | **Dilation** | **Perivasal flogosis** | **Casts** |  |  |
|  |  | **Range (0-5)** | **Range (0-4)** |  | **Range (0-4)** | **Range (0-5)** | **Range (0-4)** |  |  |
| NZB/WF1 |  | | | | | | | | |
|  | **1** | **2** | **1** | **3** | **0** | **3** | **0** | **3** | **27** |
|  | **1** | **1** | **1** | **2** | **0** | **3** | **0** | **3** | **27** |
|  | **1** | **5** | **3** | **8** | **1** | **3** | **1** | **5** | **27** |
|  | **2** | **2** | **1** | **3** | **0** | **4** | **0** | **4** | **27** |
|  | **2** | **2** | **2** | **4** | **1** | **3** | **0** | **4** | **27** |
|  | **2** | **3** | **1** | **4** | **0** | **4** | **0** | **4** | **27** |
| MRL/llpr |  | | | | | | | | |
|  | **5** | **2** | **2** | **4** | **0** | **4** | **0** | **4** | **13** |
|  | **6** | **1** | **1** | **2** | **0** | **1** | **0** | **1** | **13** |
|  | **5** | **2** | **2** | **4** | **0** | **4** | **0** | **4** | **13** |
|  | **5** | **2** | **1** | **3** | **0** | **5** | **0** | **5** | **13** |
|  | **6** | **3** | **2** | **5** | **0** | **3** | **0** | **3** | **13** |
|  | **6** | **1** | **1** | **2** | **0** | **2** | **0** | **2** | **13** |
|  | **5** | **4** | **3** | **7** | **1** | **4** | **0** | **5** | **16** |
|  | **6** | **3** | **3** | **6** | **4** | **5** | **3** | **12** | **16** |
|  | **5** | **4** | **3** | **7** | **1** | **5** | **0** | **6** | **16** |
|  | **6** | **4** | **4** | **8** | **2** | **5** | **1** | **8** | **16** |
|  | **5** | **3** | **3** | **6** | **1** | **5** | **0** | **6** | **18** |
|  | **5** | **3** | **3** | **6** | **0** | **5** | **0** | **5** | **18** |

**Supplementary Table 2. Actual histological scores of sacrificed mice**

NZB/W F1: New Zealand Black/White F1
